# *Notes from the Field:* Investigation of Colorado Tick Fever Virus Disease Cases — Oregon, 2018

**DOI:** 10.15585/mmwr.mm6812a4

**Published:** 2019-03-29

**Authors:** Emily McDonald, Debbie George, Steven Rekant, Emily Curren, Emilio DeBess, Katrina Hedberg, Jon Lutz, Jennifer Faith, Heather Kaisner, Richard Fawcett, Rebecca Sherer, Rob Kanyuch, Audrey Gudmundsson, Nicole Gardner, Marianne Salt, Olga Kosoy, Jason Velez, Erin Staples, Marc Fischer, Carolyn Gould

**Affiliations:** ^1^Arboviral Diseases Branch, National Center for Emerging and Zoonotic Infectious Diseases, CDC; ^2^Epidemic Intelligence Service, CDC; ^3^Deschutes County Health Department, Bend, Oregon; ^4^Oregon Health Authority; ^5^Bend Memorial Clinic, Bend, Oregon; ^6^St. Charles Health System, Bend, Oregon.

In early summer 2018, four cases of Colorado tick fever (CTF) were reported in residents of central Oregon; CTF virus infection was confirmed using CDC’s reverse transcription–polymerase chain reaction (RT-PCR) assay (*1*). CTF is caused by a coltivirus that is transmitted by infected Rocky Mountain wood ticks (*Dermacentor andersoni*) (*2*). The tick is found throughout the western United States and Canada, typically at 4,000–10,000 feet (1,219–3,048 meters) above sea level in grassy areas near sage brush (*3*). CTF virus causes an acute febrile illness with nonspecific symptoms, and although fatal cases are rare, up to 30% of persons with CTF virus disease require hospitalization (*4*). Because there is no definitive treatment for CTF virus disease, clinical management is supportive. Biphasic illness pattern, leukopenia, absence of rash, and place of exposure can help distinguish CTF from other arthropod-borne infections (*2*,*5*). CTF is a reportable condition in six states, including Oregon, but is not nationally notifiable. Over the past decade, the Oregon Health Authority has reported an average of less than one case of CTF per year.

CDC and Oregon health officials conducted an investigation to describe the clinical course, exposures, and geographic distribution of patients with confirmed CTF and to identify additional cases. Information was collected through medical record review and phone interview.

Three of the four confirmed cases were in men in their 70s, and one was in a woman in her 50s. The four patients were residents of three neighboring counties, and all accessed care at the same health care system in one county. Symptom onset in all four patients was in May, and all had fever, leukopenia (white blood cell count <4.0 x 10^3^/*μ*L), and thrombocytopenia (platelet count <150 x 10^3^/*μ*L). Three patients reported experiencing a biphasic illness, where their initial fever and symptoms diminished and then returned again a few days later. Three patients were hospitalized (range 1–3 days), and all recovered from their illness. Although diagnostic testing for tickborne pathogens varied, all patients were tested for CTF using RT-PCR because this test is more sensitive than serology during the acute phase of infection. All patients were treated empirically with doxycycline before laboratory confirmation of CTF virus infection.

All patients reported spending ≥5 hours per day outdoors, including working in wooded or brushy areas, and all reported a tick bite in the 2 weeks preceding illness onset. Three patients reported known tick exposures in two of the counties at elevations of 3,200–4,500 feet (975–1,372 meters) above sea level; however, no geographic clustering was identified because the land area separating the three reported tick exposure locations covered approximately 540 square miles (1,399 square kilometers). All patients reported wearing long sleeves and pants during outdoor activities, but none used insect repellent.

Electronic medical records from the same health care system as that used by the patients with confirmed cases were searched using the *International Classification of Diseases, Tenth Revision* codes for fever and leukopenia to identify possible additional cases. A suspected CTF case was defined as fever and leukopenia with no alternative explanation in a patient evaluated during April 15–July 31, 2018. Patients with suspected cases or their caregivers were interviewed and offered CTF virus testing. Three suspected CTF cases were identified in two children and one adult. The adult, a male in his 60s, submitted a serum sample that was positive for CTF virus–specific neutralizing antibodies. He acquired a tick bite in the days preceding illness onset while hunting in the same county of exposure as two of the confirmed cases.

More CTF cases were identified in Oregon in 2018 than in previous years, possibly because of increased tick activity or heightened provider awareness and testing. No common locations of tick exposure were identified, indicating the pathogen was circulating in several areas of central Oregon in spring 2018. Health departments need to reinforce tick prevention measures, including use of EPA-registered insect repellents, and target messaging to persons participating in outdoor activities with high risk for tick exposure (*6*).
